# Laparoscopic Dismembered Pyeloplasty in a Solitary Kidney with Intrarenal Pelvis: Two Challenges in One Case

**DOI:** 10.1155/2017/8169208

**Published:** 2017-11-26

**Authors:** Akin Soner Amasyali, Erhan Ates, Hakan Görkem Kazici, Alper Nesip Manav, Haluk Erol

**Affiliations:** Department of Urology, School of Medicine, Adnan Menderes University, Aydın, Turkey

## Abstract

Laparoscopic pyeloplasty (LP) for ureteropelvic junction obstruction (UPJO) is one of the most appropriate surgical techniques to achieve the optimal goal of minimally invasive surgery. However, urologists hesitate to use the laparoscopic approach in UPJO with solitary kidney or intrarenal pelvis. There are a few published studies on laparoscopic pyeloplasty cases in intrarenal pelvis. However, to the best of our knowledge, the present case is the first in the literature in terms of intrarenal pelvis in a solitary kidney. Generally, YV plasty is the accepted technique instead of dismembered pyeloplasty in UPJO with small or intrarenal pelvis. However, in this report, we showed that dismembered LP can be performed with good results in intrarenal pelvis UPJO, even if it is in the solitary kidney.

## 1. Introduction

Laparoscopic pyeloplasty (LP) was first performed by Schuessler et al. in 1993 [1]. Currently, this minimally invasive procedure has become the most common surgical approach for ureteropelvic junction obstruction (UPJO). The other minimally invasive endoscopic surgery, endopyelotomy, has lower success rates in the long term, making LP even more prominent over endopyelotomy [2]. There are different techniques for LP, such as the Anderson-Hynes dismembered pyeloplasty, fingerplasty, and YV plasty. The dismembered technique is the most preferred one and has higher success rates when compared to others. Generally, the dismembered technique is the optimal option for UPJO with extrarenal pelvis [3]. On the other hand, it is accepted that YV plasty should be preferred in patients with intrarenal pelvis [4]. In this report, we discuss a challenging case in which dismembered LP was performed in a patient who had a solitary kidney with intrarenal pelvis.

## 2. Case Report

**Figure 1:**
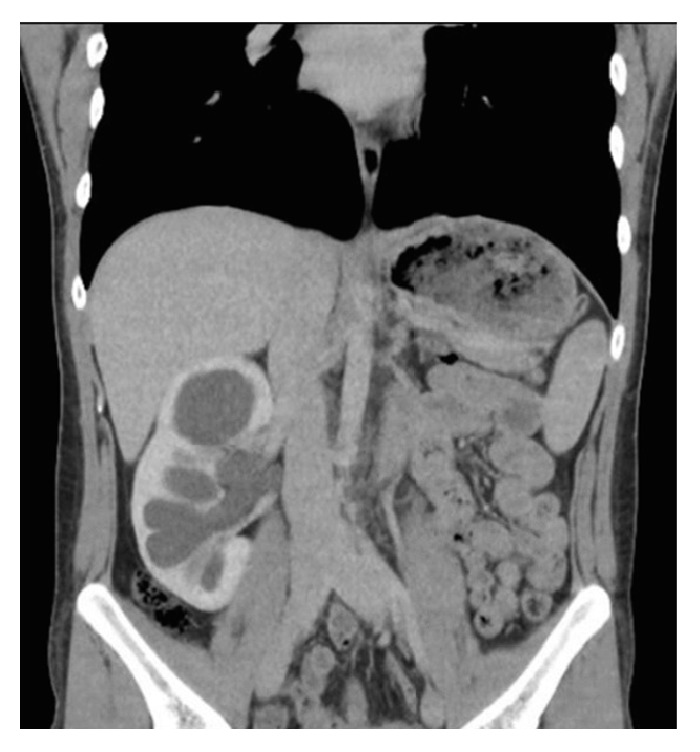
UPJO in solitary kidney with intrarenal pelvis in urography images by CT scan.

**Figure 2:**
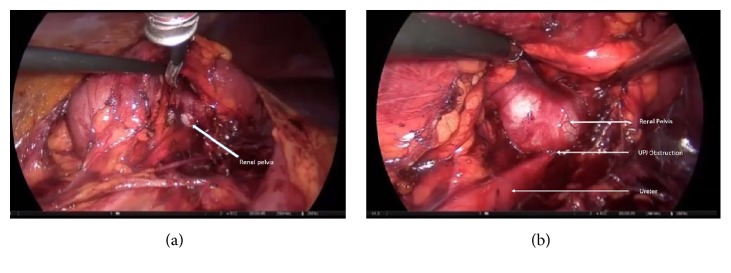
Exposure of the intrarenal pelvis and relatively high insertion of the ureter.

A twenty-three-year-old man was admitted to our outpatient clinic with right flank pain. Right costovertebral angle tenderness was positive on physical examination. Laboratory tests revealed an increased creatinin level at 1.2 mg/dL. Urinary tract imaging with ultrasonography showed grade 3 hydronephrosis in right solitary kidney. UPJO in solitary kidney with intrarenal pelvis was detected in urography images by CT scan (Figure 1). Diagnosis was confirmed by scintigraphic examination in which the urine was not able to pass through the ureter after diuretic injection. Informed consent was obtained for LP after providing information about the operation to the patient. The operation was performed transperitoneally using 4 trocars, one of which was 5 mm in diameter and the others were 10 mm in diameter. After lower pole mobilization, the ureter was dissected through the renal pelvis. When the kidney was lifted up, the intrarenal pelvis was exposed, and a relatively high insertion of the ureter was observed (Figures 2(a) and 2(b)). The dismembered technique was performed for UPJ reconstruction, and a DJ catheter was inserted antegradely. The operation was completed without any complications. Insufflation time was 240 minutes. The patient was discharged on postoperative day 3 with a creatinin level of 0.8 mg/dL.

The Double-J catheter was removed at 6 weeks postoperatively. The patient was evaluated by DTPA renography at 3 months after the operation, which revealed no evidence of obstruction. The patient had no symptoms at both follow-up visits, and creatinin levels were in the normal range (0.8 mg/dL).

## 3. Discussion

Pyeloplasty is the gold standard management option for UPJO and is indicated in deteriorated renal function, recurrent urinary tract infection, nephrolithiasis, pain, and concomitant hypertension [5]. In recent years, minimally invasive surgeries, such as laparoscopic or robotic pyeloplasty, have been more preferable than the open technique. Although a longer operation time was reported in LP, it is the most commonly used surgical approach because of shorter hospital stay, recovery time, and returning time to normal life. Good cosmetic results and a requirement of less pain control are the other advantages of LP [6].

The dismembered technique has been considered superior to nondismembering techniques due to the accurate elimination of intrinsic factors with a success rate of >90% [7, 8]. Dismembered LP is a more suitable procedure for UPJO, especially with extrarenal pelvis, due to the good exposure, easy mobilization, and adequate pelvic tissue for reconstruction after excision of the UPJ [6]. However, it is not generally recommended in intrarenal pelvis, and YV plasty is the accepted technique in this particular patient [4].

In our case, identification and mobilization of the renal pelvis and UPJ were difficult because of intrarenal pelvis localization. Usually, we perform LP with 3 ports, but in this case, we inserted a fourth trocar for lifting the kidney into an upright position. This maneuver provided us with easy access to the UPJ and mobilization of the renal pelvis. For avoiding renal parenchymal injury, a meticulous slow dissection was performed, which led to a relatively long operation time. Szydelko et al. found shorter operation times using nondismembered YV plasty compared to the dismembered technique [9]. Similar success rates were reported in their series as well. On the other hand, Casale et al. noted an overwhelming superiority of the dismembered technique over the nondismembered technique, with a success rate of 94% and 43%, respectively [10]. Finally, Lintula and Kokki reported a successful outcome in a case in which dismembered LP had been performed for UPJO with intrarenal pelvis [11]. They performed a UPJ reconstruction of a well-vascularized anastomosis, which was free of tension.

We showed that the intrarenal pelvis and high insertion of the ureter are not contraindications for dismembered LP. Dismembered LP should be considered a treatment option for UPJO in intrarenal pelvis, even if it is in a solitary kidney.
